# Major 5′terminally deleted enterovirus populations modulate type I IFN response in acute myocarditis patients and in human cultured cardiomyocytes

**DOI:** 10.1038/s41598-020-67648-5

**Published:** 2020-07-20

**Authors:** M. Glenet, Y. N’Guyen, A. Mirand, C. Henquell, A.-L. Lebreil, F. Berri, F. Bani-Sadr, B. Lina, I. Schuffenecker, L. Andreoletti, The French Enterovirus Myocarditis Study Group (FEMSG), A. Mirand, C. Henquell, Marie-Laure Mathieu, Ellia Mezgueldi, Matthieu Verdan, Pascal Motreff, B. Lina, I. Schuffenecker, Samira Fafi-Kremer, Quentin Lepiller, Patrick Bruneval

**Affiliations:** 1 University of Reims Champagne-Ardenne and EA4684 Cardiovir Research Laboratory, Reims, France; 2 Centre Hospitalier Universitaire de Reims, Reims, France; 3 National Reference Center of Enterovirus and Parechovirus, Clermont-Ferrand, France; 4 University of Clermont Auvergne, Clermont-Ferrand, France; 5 Centre Hospitalier Universitaire Clermont-Ferrand, Clermont-Ferrand, France; 6 National Reference Center of Enterovirus and Parechovirus, Lyon, France; 7 Hospices Civils de Lyon, Lyon, France; 8 University of Lyon, Lyon, France; 9 Hôpital Européen Georges Pompidou (HEGP), Paris University Hospital, AP-HP, Paris, France

**Keywords:** Cardiology, Pathogenesis

## Abstract

Major 5′terminally deleted (5′TD) group-B enterovirus (EV-B) populations were identified in heart biopsies of patients with fulminant myocarditis or dilated cardiomyopathy suggesting that these 5′TD forms are key drivers of host-cell interaction in EV cardiac infections. To date, early emergence of EV-B 5′TD forms and its impact on type 1 IFN response during acute myocarditis remains unknown. Using quantitative RACE-PCR assay, we identified major EV-B 5′TD RNA populations in plasma or heart samples of acute myocarditis cases. Deletions identified within the 5′ non-coding region of EV-B populations only affected secondary-structural elements of genomic RNA domain I and were distinguished in two major groups based on the extent of RNA structural deletions. Proportions of these two respective EV-B 5′TD population groups were positively or negatively correlated with IFN-β levels in plasma samples of myocarditis patients. Transfection of synthetic CVB3/28 RNAs harboring various 5′terminal full-length or deleted sequences into human cultured cardiomyocytes demonstrated that viral genomic RNA domain I possessed essential immunomodulatory secondary-structural elements responsible for IFN-β pathway induction. Overall, our results highlight the early emergence of major EVB-TD populations which deletions affecting secondary–structures of RNA domain I can modulate innate immune sensing mechanisms in cardiomyocytes of patients with acute myocarditis.

## Introduction

Group-B Enteroviruses (EV-B) belong to the *Picornaviridae* family and are recognized as major causes of aseptic meningitis, upper or lower respiratory tract infections and acute myocarditis cases diagnosed in neonates, infants and young adults^1,2^. Between 10–20% of acute myocarditis cases will evolve into chronic myocarditis as well as dilated cardiomyopathy (DCM, prevalence = 7 cases / 100,000, second leading cause of heart transplantation worldwide after ischemic heart disease)^1^.

EV-B RNA genome is approximately 7,400 nucleotides (nt) in length and is flanked at the 5′ end by a highly conserved non-coding region (5′NCR) that is crucial for the initiation of the viral replication and translation activities^3^. A study in 2008 of heart tissue from a patient who died of fulminant myocarditis demonstrated the presence of a group B Coxsackie virus type 2 (CVB-2) population with partial deletions at the 5′ terminus and proceeding inward^4^. Similar findings had been described following CVB-3 replication in cell cultures and in mice^5,6^. Bouin et al.^7^ identified EV-B populations characterized by 5′NCR RNA deletions ranging from 15 to 48 nt either alone or associated with low proportions of intact 5′NCR termini in heart biopsies collected from a living woman suffering from unexplained DCM^7^. These results were subsequently extended by NGS (Next Generation Sequencing) investigation of explanted heart tissues from a cohort of unexplained DCM adult patients^8^. Together, these findings suggested that EV-B populations with 5′ terminally deleted (5′TD) genomes can be generated and selected in heart tissues during the early acute viral myocarditis. The emergence of these low replicative TD populations offer a valid explanation on how EV-B can persist in hearts long after the acute infection during the chronic myocarditis of the clinical phase of DCM.

Natural deletions within the 5′NCR of EV-B RNA populations can affect functional secondary-structural elements of the RNA domain I (cloverleaf, CL)^9–11^ altering formation or stability of viral replication complexes and consequently decreasing genomic replicative capacities of 5′TD viral RNA forms^12–14^. Moreover, it was evidenced that the 5′triphosphorylated cloverleaf structure of CVB3 RNA is sufficient to induce a type 1 IFN response by the RIG-I signalling pathway^10^. In addition, it was reported that complete double-strand RNA replicative forms of EV-B were recognized by MDA5 triggering a significant type 1 IFN response in cultured human or mice cells^9^. Early natural deletions into the 5′NCR of EV-B populations could modify the RNA secondary-structural elements recognized by cytoplasmic immune sensors, such as RIG-I or MDA5 (RLRs, RIG-I-Like Receptors), resulting in a modulation of type 1 IFN pathway induction in human cells, a potential way to overcome antiviral immune innate host response leading to persistent infection in target tissues^15^. To date, the impact of 5′TD viral RNA forms on viral replication activities and type 1 IFN response levels remain to be fully investigated in EV-B induced acute myocarditis.

In the present report, using a quantitative RACE-PCR system we identified major EV-B 5′TD RNA populations associated with minor full-length (FL) viruses in peripheral blood or heart tissue samples of acute myocarditis pediatric patients. Identified deletion groups within the 5′NCR of major EV-B RNA populations affected only secondary-structural elements of the RNA domain I (cloverleaf). Using correlation analyses, we assessed the impact of EV-B 5′TD population proportions on viral genomic replication activities and type 1 IFN (interferon beta, IFN-β) secretion levels in peripheral blood samples of myocarditis patients. Subsequently, transfection of synthetic CVB3/28 RNAs harboring various 5′terminal full-length or deleted sequences were generated in vitro and their impact on type 1 IFN pathway induction was assessed on HeLa229 and human primary cardiomyocytes (HCM) cultures.

## Results

### Identification of group B Enteroviruses in heart tissues or peripheral blood samples of acute myocarditis patients

**Figure 1:**
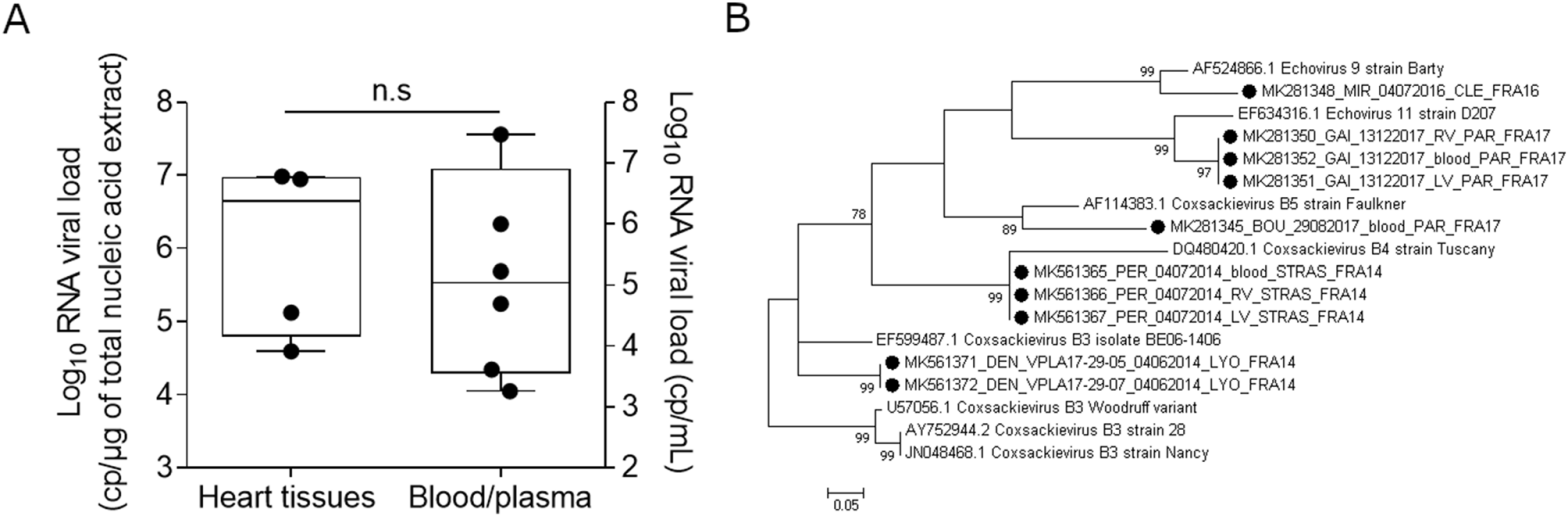
Enterovirus-RNA genome levels and phylogenetic viral VP1 gene sequences obtained from heart tissues or peripheral blood samples from acute myocarditis patients. (**A**) Viral RNA genomic replication activity was assessed using a RT-qPCR assay on heart samples (copies/ng) (n = 4) or blood and plasma samples (copies/mL) (n = 6). Data represent the median with range. Non-significant (n.s) according to Mann–Whitney U test. (**B**) Molecular phylogenetic analysis (nucleotide comparative analysis) of the enterovirus VP1 gene was inferred by using the maximum likelihood method with MEGA version 7.0. A total of 1,000 bootstrap iteration has been calculated to assess the toughness of the method, percentage of trees in which the associated taxa clustered together is shown next to the branches. The scale refers to the distance between sequences. Black dots represent the eleven identified strains isolated during the present study. GenBank accession nos. of the eleven identified strains are the following: MK281350-MK281351-MK281352-MK281348-MK281345-MK561365-MK561366-MK561367-MK561371- MK561372).

Among the study patients [5 males/1 female; median age 21.6 days (3.6—90 days)] median time delay between symptoms and sample collection was 3.58 days (1—7 days) (Table 1). An EV-genus specific real-time quantitative reverse transcription PCR protocol demonstrated an endomyocardial standardized median viral genome load value of 4.50 × 10^6^ copies [range 3.97 × 10^4^–9.65 × 10^6^] per microgram of total extracted nucleic acids (Fig. 1A) and a tenfold lower median EV viral load value of 1.09 × 10^5^ copies [range 1.82 × 10^3^–2.98 × 10^7^] per mL of plasma in study acute myocarditis patients (Fig. 1A). Phylogenetic analysis and genotypic identification were based on the partial viral protein 1 (VP1) nucleotide sequence using reverse transcription PCR of the VP1 into 10 peripheral blood or biopsy heart samples taken from 6 patients (Fig. 1B). All strains of clinical samples were classified as group B Enterovirus (EV-B) strain and we identified five different species in study patients. As displayed in Fig. 1B, four patients were clustered with group B coxsackieviruses (CVB-3, CVB-4 and CVB-5) and two patients grouped into Echovirus 9 and 11 clusters.

**Table 1:** Demographic, clinical and virological data in study acute myocarditis patients.

*Demographic data*	
Number of patients with myocarditis/Number of samples (n=)	6/10
Median age (days)	21.6 [3.6–90]
Sex (males/female)	5/1
*Clinical data*	
Blood samples (%)	6 (60)
Hearts samples (autoptic) (%)	4 (40)
Median delay between beginning of symptoms and time of hospitalization (days)	3.58 [1–7]
*Virological data*	
Median EV RNA level detection (blood samples)(genomes copies/ml)	1.09x10^5^ [1.82x10^3^–2.98x10^7^]
Median EV RNA level detection (heart samples) (genomes copies/μg)	4.5x10^6^ [3.97x10^4^–9.65x10^6^]
Proportion of full-length form (FL) (%)	7 [0–18.0]
Proportion of 15 to 36 nt TD forms (%)	3 [0–15.0]
Proportion of full-length and 15 to 36 nt TD forms (%)	10 [0–18.0]
Proportion of 37 to 50 nt TD forms (%)	90 [66.97–94.95]

Values are expressed as median; EV, enterovirus.

### Quantitative detection of EV-B 5′terminally deleted RNA populations using a RACE-PCR system

**Figure 2:**
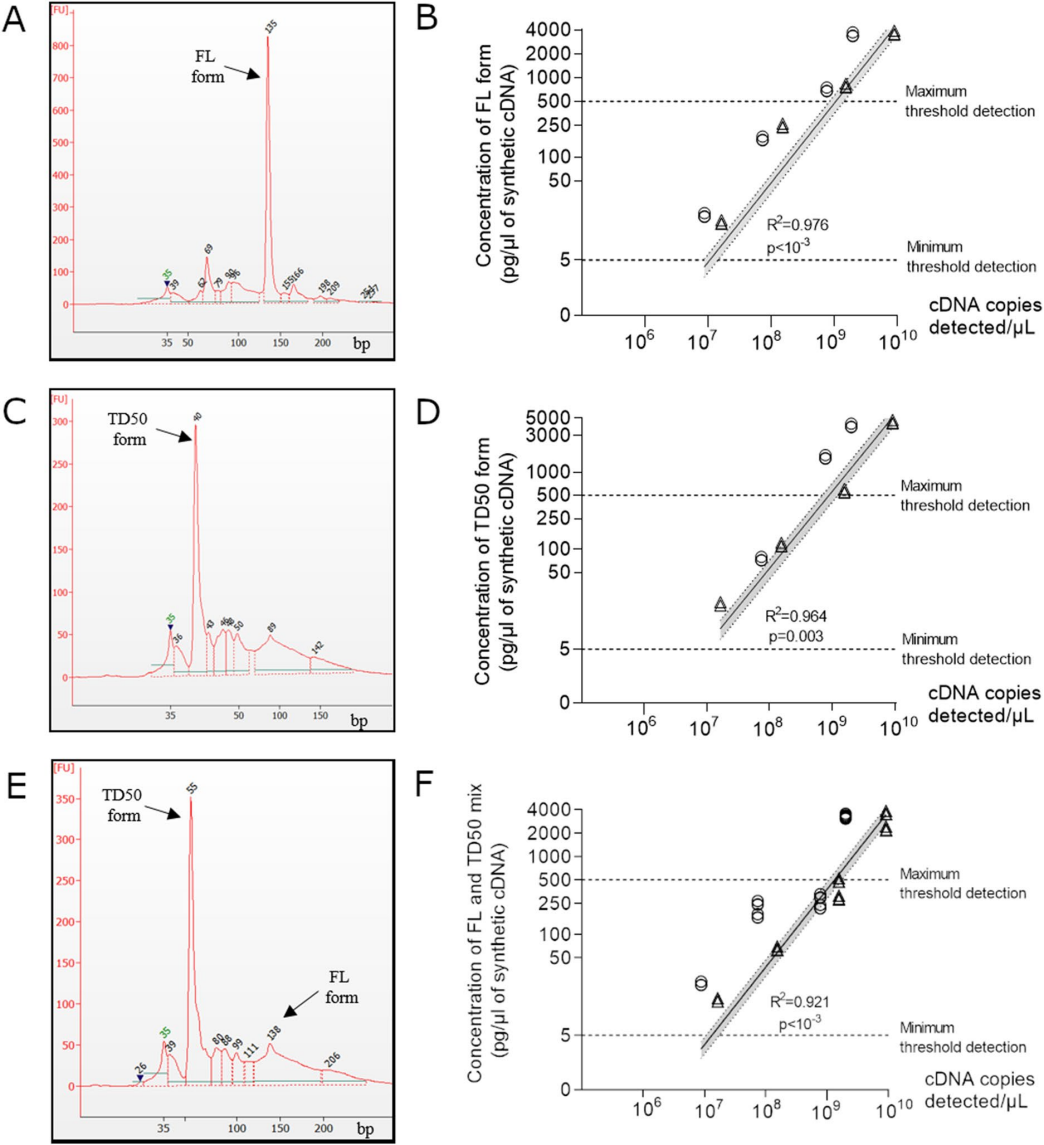
Quantitative detection of undeleted and 5′terminally deleted EV-B populations in clinical samples. (**A**) Electrophoregrams of RACE-PCR FL synthetic cDNA (135 bp ± 10%). (**B**) Quantification of EV-B full-length (FL) form after serial tenfold dilutions of synthetic FL form diluted in human cardiac (○) or plasmatic (∆) total RNA extracts of EV-negative human samples (n = 3). Linear regression curve between cDNA copies detected per µL and concentration of full-length synthetic cDNA. (**C**) Electrophoregrams of TD50 synthetic cDNA (55 bp ± 10%) after RACE-PCR. Image obtained from “Agilent 2,100 Bioanalyzer” after analysis with High sensitivity DNA (Agilent). (**D**) Quantification of EV-B 5′terminally deleted of 50 nt (TD50) form after serial tenfold dilutions of synthetic TD50 form diluted in human cardiac (○) or plasmatic (∆) total RNA extracts of EV-negative human samples (n = 3). Linear regression curve between cDNA copies detected per µL and concentration of TD50 form of synthetic cDNA. (**E**) Electrophoregrams of FL and TD50 mix (95% of TD50 form with 5% of FL form) synthetic cDNA after RACE-PCR. Image obtained from “Agilent 2,100 Bioanalyzer” after analysis with High sensitivity DNA (Agilent). (**F**) Quantification of EV-B FL and TD50 mix (95% of TD50 form with 5% of FL form) forms after serial tenfold dilutions of synthetic FL and TD50 synthetic forms diluted in human cardiac (○) or plasmatic (∆) total RNA extracts of EV-negative human samples (n = 3). Linear regression curve between cDNA copies detected per µL and concentration of mix FL and TD50 form of synthetic cDNA. The dashed lines indicate the thresholds of detection. Linear regression was performed, and slopes were compared using Spearman test. FL, full-length; TD, terminally deleted, bp, base paired.

To detect and quantify EV-B RNA of FL as well as 5′TD forms in cardiac tissue samples and peripheral blood samples from acute myocarditis patients, we used a sensitive and specific rapid amplification of cDNA ends (RACE) PCR followed by micro-electrophoresis assay (Supplementary Figure S2)^7^. Our RACE-PCR assay allowed to size viral cDNA with 5′deletions ranging from 35 to 137 bp with an accuracy of 10% established by manufacturer, even in cases of mixed RNA forms (Fig. 2A,C,E). Concentrations of synthetic FL, 50 nt deleted (TD50) viral or mixed FL and TD50 cDNA forms diluted in plasmatic or cardiac cDNA appeared to be positively correlated with estimated cDNA copies per µl of PCR product using our RACE-PCR strategy (R^2^ = 0.976, *P* < 10^–3^ for FL form; R^2^ = 0.964, P = 0.003 for TD50 form and R^2^ = 0.921, *P* < 10^–3^ for FL and TD50 mix) (Fig. 2B,D,F).

**Figure 3:**
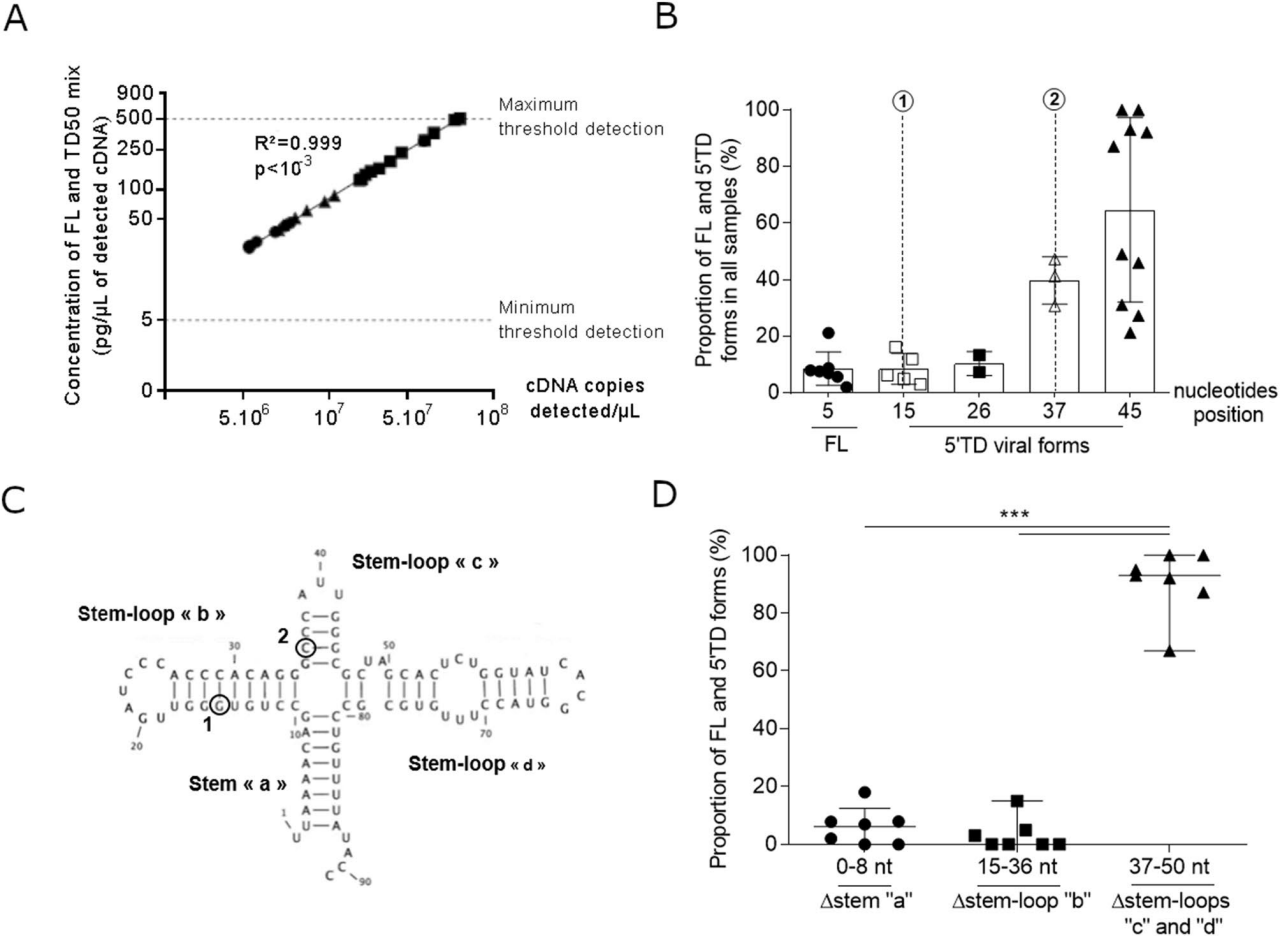
Detection of full-length and 5′terminally deleted EV-B population levels in heart and peripheral blood samples. (**A**) Linear regression curve between cDNA copies detected per µL and concentration of FL and 5′TD forms of detected cDNA in heart and peripheral blood samples (n = 10). Linear regression was performed, and slopes were compared using Spearman test. (**B**) Identification of FL and 5′TD EV-B and proportion of each 5′TD forms of study samples. (**C**) Two-dimensional representation of the EV-B 5′non-coding region (NCR) RNA sequences inducing a cloverleaf replant with a stem “a”, and three stem-loop “b” to “d” essential for EV-B replication. In position 1, the position of the EV-B 5′ terminally deleted forms with 15 nucleotides. In position 2, the position of the EV-B 5′TD forms with 37 nucleotides. (**D**) Proportion of 5′TD forms (15–36 nt and 37–50 nt) and FL form in heart and peripheral blood samples. Data represent the mean ± SD. Viral populations deleted from less than 8 nucleotides were considered as full- length viral populations. FL, full-length; TD, terminally deleted, bp, base paired, nt, nucleotides.

Using our RACE-PCR system, we assessed the concentrations and the respective proportions of EV-B FL and 5′TD forms in heart tissue and peripheral blood samples taken from patients with acute myocarditis (Fig. 3 and Supplementary Figure S3). Concentrations of FL and various 5′TD forms appeared to be statistically well-correlated with estimated cDNA copies per µl of PCR product validating our quantitative RACE-PCR approach (R^2^ = 0.999, *P* < 10^–3^) (Fig. 3A). We identified various 5′TD viral forms with deletion ranging from 5 to 45 nt which proportion levels are displayed in Fig. 3B. Viral populations deleted from less than 8 nt were considered as full-length (FL). Various identified deletions at the terminus of the 5′NCR of viral RNA affect the secondary structure, resulting in the loss of stem “a”, stem-loop “b”, “c” and part of stem-loop “d” of the cloverleaf structure (Fig. 3B,C). Taking into account the secondary structure of the EV-B RNA cloverleaf structure, these 5′ TD viral populations can be distinguished as three subgroups based on the extent of RNA deletions: (i) TDs ranging from 0 to 8 nt resulting in the loss of stem “a”, (ii) TDs ranging from 15 to 36 nt resulting in the loss of stem “a”, stem-loop “b,” and (iii) TDs ranging from 37 to 50 nt resulting in the loss of stem “a”, stem-loop “b”, “c” and part of stem-loop “d” in the 5′ cloverleaf RNA structure.

Finally, our molecular approach identified major EV-B populations characterized by 5′TD population ranging between 37–50 nt (90%) in length which was associated with minor proportions of 5′TD 15–36 nt (3%) and FL RNA forms (7%) (Fig. 3D).

### Impact of EV-B 5′terminally deleted populations on viral genomic replication activities in plasma of acute myocarditis patients

**Figure 4:**
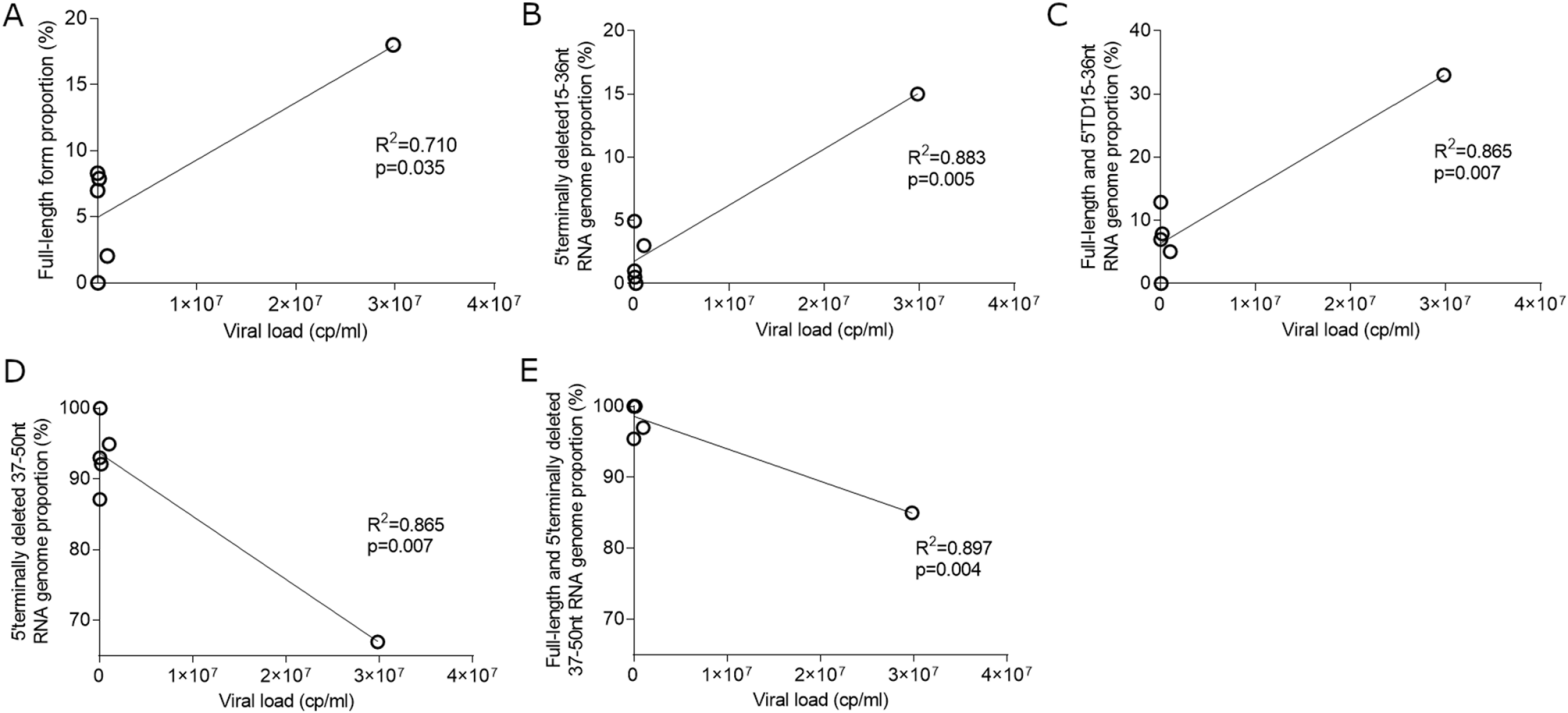
Relationships between EV-B full-length or 5′terminally deleted RNA population proportions and total viral RNA levels in peripheral blood of acute myocarditis patients. (**A**) FL form proportion (%) correlation between EV-B viral load (cp/mL) (n = 6). (**B**) 5′terminally deleted 15–36 nt RNA genome proportion (%) correlation between EV-B viral load (cp/mL) (n = 6). (**C**) Full-length associated with 5′terminally deleted 15–36 nt RNA genome proportion (%) correlation between EV-B viral load (cp/mL) (n = 6). (**D**) 5′terminally deleted 37–50 nt RNA genome proportion (%) correlation between EV-B viral load (cp/mL) (n = 6). (**E**) Full-length associated with 5′terminally deleted 37–50 nt RNA genome proportion (%) correlation between EV-B viral load (cp/mL) (n = 6). Linear regression was performed, and slopes were compared using Spearman test. FL, full-length; TD, terminally deleted.

To assess whether 5′TD RNA populations levels could modulate viral genomic replication activities, 5′TD viral RNA form groups proportions were compared with total EV viral RNA loads measured in plasma samples (Fig. 4). A positive correlation was observed between associated 5′TD 15–36 nt and FL RNA forms proportions and EV total RNA levels (R^2^ = 0.865; P = 0.007) (Fig. 4C). Interestingly, the proportions of FL or 15–36 nt deleted EV-B RNA forms alone appeared to be positively correlated with EV total RNA levels (FL: R^2^ = 0.710; P = 0.035 and 15–36 nt deleted forms: R^2^ = 0.883; *P* = 0.005) (Fig. 4A,B). By contrast, a negative correlation was observed between EV total RNA levels and the proportion of 37–50 nt deleted EV-B RNA forms (R^2^ = 0.865, *P* = 0.007) (Fig. 4D). Moreover, a negative correlation was observed between EV total RNA levels and minor FL population associated to 37–50 nt deleted EV-B RNA forms proportions (R^2^ = 0.897, *P* = 0.004) (Fig. 4E). These findings suggested the impact of the extent of RNA deletions in the 5′cloverleaf RNA structure on the viral genomic replication levels in peripheral blood of acute myocarditis patients.

### Impact of EV-B 5′terminally deleted populations on type 1 IFN levels in plasma of acute myocarditis patients

**Figure 5:**
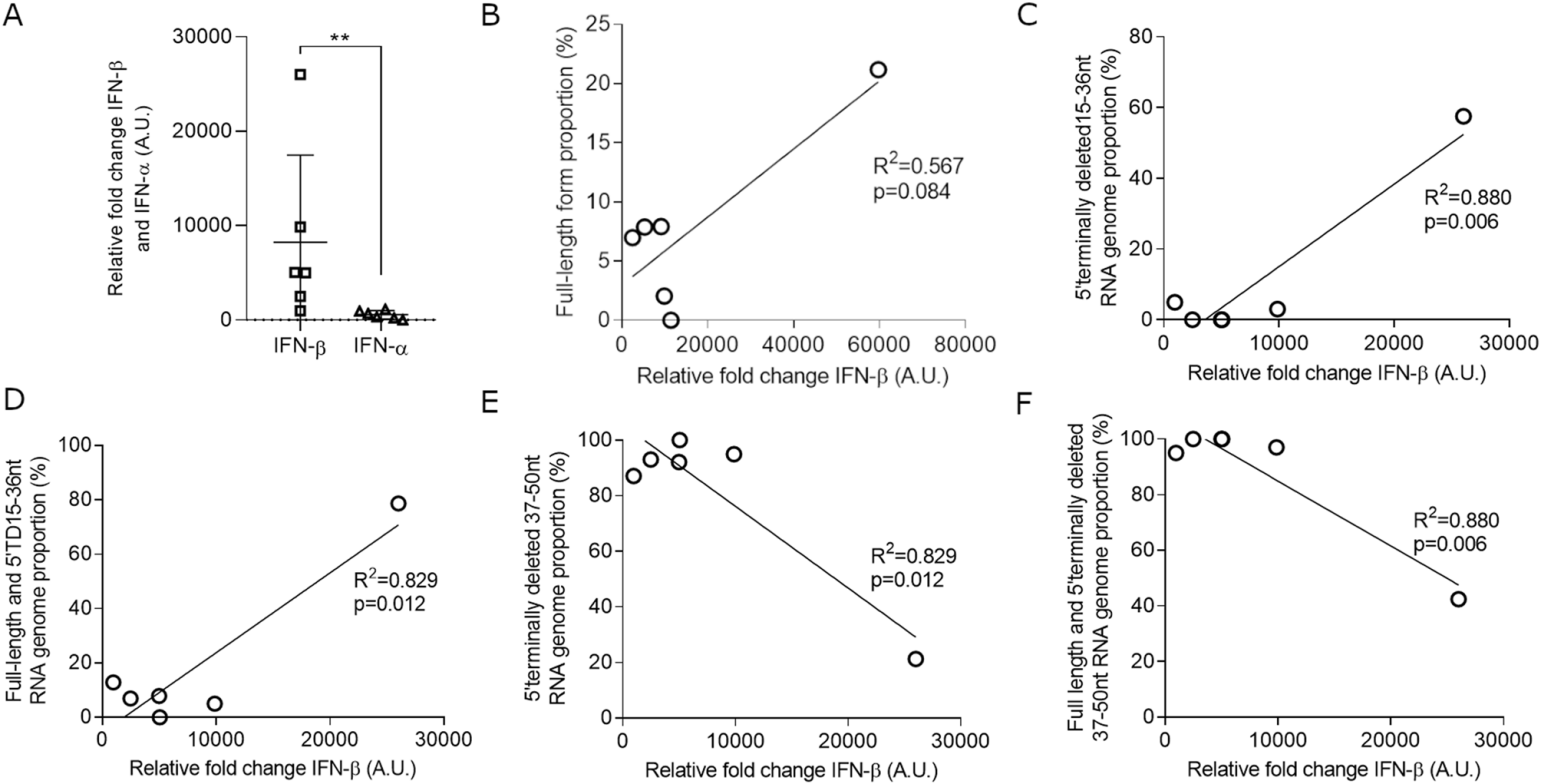
Relationships between EV-B full-length or identified EV-B 5′terminally deleted population proportions and type 1 IFN levels. (**A**) IFN-α and IFN-β semi-quantitative RT-qPCR analysis in peripheral blood samples expressed as fold change over control patients after normalization to housekeeping mRNA expression. Data represent the mean ± SD. **P < 0.01 according to Mann–Whitney U test. (**B**) FL form proportion (%) correlation between relative fold change of IFN-β (A.U.) (n = 6). (**C**) 5′terminally deleted 15–36 nt RNA genome proportion (%) correlation between relative fold change of IFN-β (A.U.) (n = 6). (**D**) Full-length and 5′terminally deleted 15–36 nt RNA genome proportion (%) correlation between relative fold change of IFN-β (A.U.) (n = 6). (**E**) 5′terminally deleted 37–50 nt RNA genome proportion (%) correlation between relative fold change of IFN-β (A.U.) (n = 6). (**F**) Full-length and 5′terminally deleted 37–50 nt RNA genome proportion (%) correlation between relative fold change of IFN-β (A.U.) (n = 6). Linear regression were performed, and slopes were compared using Spearman test. FL, full-length; TD, terminally deleted.

Using correlation analyses, we investigated the impact of identified EV-B 5′TD population proportions on type 1 IFN mRNA levels in peripheral blood of acute myocarditis patients (Fig. 5). Interestingly IFN-β mRNA levels appeared significantly higher to IFN-α mRNA in study patients (Fig. 5A). No correlation was observed between relative fold change of IFN-α and proportions of FL, 5′TD 15–36 nt, 5′TD 37–50 nt, FL associated with 5′TD 15–36 nt or FL associated with 5′TD 37–50 nt populations (Supplementary Figure S4). No correlation was observed between FL form proportion alone and IFN-β levels (Fig. 5B). However, a positive correlation was observed between 5′TD 15–36 nt deleted EV-B RNA forms proportions alone and IFN-β levels (R^2^ = 0.880; P = 0.006) (Fig. 5C). Also a positive correlation was observed between minor FL population associated with 5′TD 15–36 nt deleted EV-B RNA forms proportions and IFN-β levels (R^2^ = 0.829; P = 0.012) (Fig. 5D). By contrast, a negative correlation was observed between 37–50 nt deleted EV-B RNA forms alone or associated with FL forms proportion and IFN-β levels (R^2^ = 0.829, P = 0.012 and R^2^ = 0.880, P = 0.006, respectively) (Figs. 5E,F). These results suggest that populations and proportions of 5′ terminal deletions of EV-B RNA forms modulate IFN-β response in acute myocarditis patients.

## Type 1 IFN pathway induction in HeLa229 or human cultured cardiomyocytes

**Figure 6:**
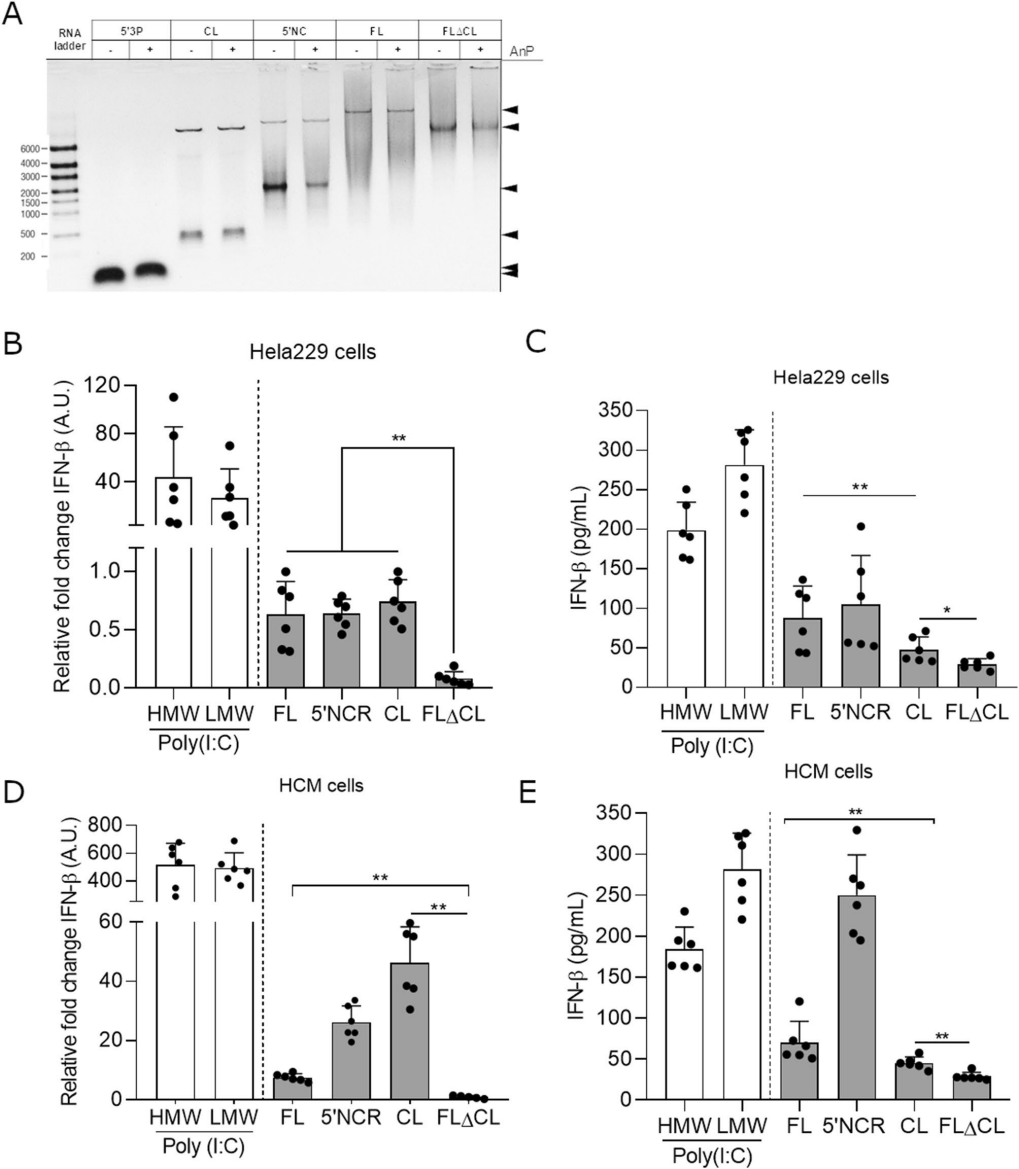
IFN-β mRNA synthesis and secretion levels by HeLa229 or human primary cardiac cells transfected by CVB3/28 full-length or 5′terminally deleted RNA forms. (**A**) Synthetic CVB3/28 RNAs 5′NC, CL, FL and TD100 were untreated or treated by Antarctic Phosphatase (AnP). Result of synthetic RNA forms treatment by AnP was visualized using 1% agarose gel electrophoresis. Arrows indicated the RNA band sizes on the gel picture. (**B**) and (**D**) IFN-β mRNA were expressed as fold change over mock transfected cells after normalization to housekeeping mRNA expression respectively in immortalized HeLa229 cells and in human primary cardiomyocytes. Data represent mean ± SD (n = 3) (Mann–Whitney U test; **P  < 0.01). (**C**) and (**E**) IFN-β levels were quantified by ELISA in supernatants of cardiomyocytes cells at 8 h’ post-transfection of various EV-B RNA forms. Data represent mean ± SD (n = 3) (Mann–Whitney U test; * *P* < 0.05 and **P < 0.01). CVB3/28 RNA, full-length form (FL) and deleted CVB3/28 forms of 100 nucleotides (TD100); Positive controls: CL (Cloverleaf, Domain I), 5′NC: 5′non-coding region; LMW (poly (I:C) low molecular weight), HMW (poly (I:C) high molecular weight).

Since EV-B RNA 5′ terminal deletions forms were associated with a modulation of IFN-β response in acute myocarditis patients, a key biological issue was to identify 5′NCR secondary-structural elements capable of regulating type 1 IFN activation pathway in human target cells (Fig. 6). Various CVB3/28 RNA sequences including full-length (FL) genomic RNA, cloverleaf (CL), 5′non-coding region (5′NCR) and FL viral RNA forms without cloverleaf (FL∆CL) were cloned and in vitro transcribed. These synthetic CVB3/28 RNA forms were positive single strand RNA structures without 5′tri-phosophate groups and were therefore unable to induce a non-specific type 1 IFN response via RIG-I pathway activation in target cells^10^ (Fig. 6A). To investigate the modulatory effect of type 1 IFN activation pathway by EV-B 5′TD viral RNA structures in human cells, transfection of our synthetic viral RNA forms was performed in HeLa229 and then in cultured primary cardiac cells (HCM). (Fig. 6B–E).

Interestingly, HCM transfection of synthetic CVB3/28 CL and 5′NCR RNA forms induced significantly higher levels of IFN-β mRNA than those observed with FL RNA forms at 8 h’ post-transfection (P < 0.01) (Fig. 6C). By contrast, IFN-β mRNA levels appeared to be significantly lower after FL∆CL transfection in HeLa229 and cardiac cells than those observed following transfection of CL, 5′NCR and FL viral RNA forms (P < 0.01) (Fig. 6B,D). IFN-β cytokine levels was quantified in the supernatant of HeLa229 and HCM cells transfected by the same synthetic RNA forms and confirmed significant variations of IFN-β mRNA levels following FL∆CL viral RNAs transfection (Fig. 6C,E). Overall, our findings indicated that EV-B 5′NCR genomic RNA domain I (cloverleaf) contained sequences or secondary structural elements responsible for IFN-β pathway induction in HeLa229 and HCM.

## Discussion

Major 5′terminally deleted (5′TD) Group-B enterovirus (EV-B) populations were previously identified in heart biopsies of patients with fulminant myocarditis or dilated cardiomyopathy suggesting that these 5′TD forms are key drivers of host-cell interaction in EV cardiac infections. In the present report, two major 5′TD RNA population groups were identified in peripheral blood or heart tissue samples of EV-B acute myocarditis cases and their respective proportions appeared to be positively or negatively correlated with type 1 IFN levels. To confirm immunomodulatory effects of these 5′TD RNA forms on the type 1 IFN pathway induction, synthetic CVB3/28 RNAs harboring various 5′terminal full-length (FL) or deleted genomic sequences were transfected into HeLa229 or human primary cardiomyocytes cultures. This in vitro approach displayed that EV-B genomic RNA domain I (Cloverleaf (CL)) possessed essential immunomodulatory secondary-structural elements responsible for IFN-β pathway induction in human target cells.

We identified major EV-B 5′TD RNA forms in rare peripheral blood or heart tissue samples taken from pediatric patients hospitalized for an EV-related acute myocarditis (Table 1 and Fig. 1). Our 5′TD RNA populations identification is consistent with, and extend the results published previously for, a fulminant human myocarditis^16^ and in a recent cohort of EV-induced DCM (Fig. 2) ^7^. In the present study focusing on acute myocarditis cases, 15 to 36nt deleted EV-B RNA forms proportions and FL RNA forms were quantified in higher proportions than those recently reported by our group in EV-induced DCM cases. These discrepancies could partly explain the different levels of EV replication observed in heart tissues between EV-related acute myocarditis and EV-related persistent cardiac infection demonstrated in chronic myocarditis and DCM cases.

Interestingly, taking into account the secondary structure of the EV-B RNA cloverleaf (RNA domain I), 5′TD viral populations were distinguished as two major groups (8–36 nt and 37–50 nt EV-B 5′TD forms) based on the extent of RNA deletions (Fig. 3). Proportions of 5′TD 15-36nt forms alone or associated with FL were positively correlated with EV-B viral load levels in peripheral blood samples, whereas 5′TD 37–50 nt forms alone or associated with FL genomic RNA were negatively correlated with peripheral EV-B RNA levels in the same peripheral plasma blood samples (Fig. 4). Such terminal 5′NCR deletions are known to affect functional secondary-structure of viral EV-B RNA domain I^9,10,17^, corresponding to a cloverleaf structure, decreasing the ability of cellular factors binding to the viral RNA replication complex and resulting in lower viral RNA replication levels in CVB3-infected cardiomyocytes ^17^. The major 5′TD 37–50 nt forms loosened a part of the 5′translated region cloverleaf structure, but the viral RNA polymerase (3CD) binding site remains intact (Fig. 3C,D). Finally, these deletions resulted in the loss of stem “a”, stem-loop “b,” stem-loop “c,” and part of stem-loop “d” in the 5′ cloverleaf RNA structure (Fig. 3C), consistent with results published previously for a fulminant human myocarditis^4^ and in a recently published case of EV-induced DCM^8^. These RNA deletions disrupt the host protein PCBP binding site located in stem-loop “b”^18^ and decrease the viral RNA replication activities and finally resulted in a negative correlation observed between the proportion of 5′TD 37–50 nt deleted viral RNA populations and total viral RNA load values (Fig. 4D). Since proportions of 37–50 nt EV-B 5′TD populations were negatively correlated with the total EV RNA levels in peripheral blood, we hypothesized that these viral forms were low-level replicating viruses which sustained genomic replication activities would result from molecular cooperation mechanisms with other viral RNA populations. Full-length or 8–36 nt EV-B 5′TD viruses could play the role of helper virus by providing, in trans or through genomic recombination events, the elements necessary for replication of 37–50 nt EV-B 5′TD RNA forms^19,20^. These 5′TD viral forms associated with 8–36 nt 5′TD or FL viruses could reproduce in vivo a mode of viral replication that was reported to occur in cell culture via defective interfering (DI) particles^21^. Further experiments based on co-transfection of synthetic CVB3/28 FL and various 5′TD RNA forms in human cultured cardiac cells are needed to explore this hypothesis.

We hypothesized that levels of minor FL and 5′TD 15–36 nt forms could positively increase EV genomic RNA replication activities and total EV viral loads in heart tissue, therefore modulating the activation pathways of inflammatory response and resulting in more severe cardiac histological injuries in acute myocarditis cases^8,17^. Interestingly, in only one study subject, the distribution of median levels of 5′TD viral forms appeared not to be significantly different between peripheral blood and heart compartments in an intra-patient comparative analysis, suggesting that peripheral blood circulating viral RNA population levels detected in the plasma blood were representative of those detected in cardiac tissues (Supplementary Figure S1). It is intriguing to consider that the quantitative detection of various minor EV-B RNA populations could be a potential peripheral blood biomarker of acute myocarditis severity (Fig. 3). Being able to quantify various mixtures of EV-B FL or 5′TD forms in plasmatic or cardiac samples RNA would allowed the evaluation of 5′TD viral forms dynamics apparition and the measurement of viral RNA levels to simplify an assessment of EV-induced acute myocarditis with normal, mild or severe cardiac histological injuries, thus helping to diminish the frequency of performing endomyocardial biopsy (EMB) in patients. Further experiments using NGS approaches of the EV-B 5′NCR RNA detected genome, will confirm the presence of major 5′TD detected in EV-B RNA forms in heart tissue or peripheral blood samples of acute myocarditis cases in pediatric and adult patients. Moreover, such NGS approaches integrated in prospective and longitudinal clinical studies would allow investigation of the potential positive predictive value of minor FL or 5′TD proportion forms in the development of severe acute or fulminant myocarditis cases.

We showed for the first time that 8–36 nt and 37–50 nt EV-B 5′TD population’s proportions could modulate IFN-β levels in peripheral blood samples of acute myocarditis patients. We observed that these two groups of 5′terminally RNA deletions differentially affected secondary–structures of RNA domain I which could result in a differential qualitative binding of EV-B 5′TD RNA sequences to the RLRs (RIG-I or MDA5) acting as major innate immune sensors during EV-B acute infections^22^. In an effort to understand the link between RNA domain I structure and IFN-β pathway induction, transfection of synthetic CVB3/28 RNAs harboring various 5′terminal FL or deleted genomic sequences was assessed into HeLa229 and then in cultured human primary cardiomyocytes. Our original results demonstrated that viral genomic RNA domain I contained essential immunomodulatory secondary-structural elements responsible for IFN-β pathway induction (Fig. 6). To date, the exact secondary RNA domain I structures recognized by RLRs (RIG-I or MDA5) possibly in a LGP2-dependant manner, remains unknown. We are currently addressing this question by generating synthetic FL forms specifically deleted within the various stem-loops of EV-B RNA domain I and using overexpressing and knock-out HEK293T cells for RLRs^22^. Modulation of RLRs activation by 5′terminal RNA structures of EV-B 5′TD viruses could impair type 1 IFN pathway activation, thus regulating IFN-β and interferon-stimulated gene (ISG) transcription and translation levels in infected cells, a potential way to overcome antiviral immune innate host response.

In summary, our results highlight the early emergence of major EV-B 5′TD populations which deletions affecting secondary–structures of RNA domain I can modulate innate immune sensing mechanisms in cardiomyocytes of patients with acute myocarditis. These results provide new insights into the role of EV-B 5′TD RNA forms on innate immune mechanisms and inflammatory responses in heart tissue and should stimulate the research for the development of new therapeutic strategies based on modulation of type 1 IFN pathway in EV-B induced acute cardiac infections.

## Methods

### Patient selection

Peripheral blood plasma (n = 6) and cardiac tissue samples (n = 4) were collected between 2014 and 2017 and stored in three biobanks (−80 °C) [Enterovirus National Reference Centers Hospices civils de Lyon; Clermont-Ferrand University Hospital Center; Hôpital Européen Georges Pompidou Paris University Hospital (AP-HP, PARIS, France)] were selected because: (i) they were collected from patients demonstrating a final recorded clinical diagnosis of acute myocarditis established according to ESC guidelines by physicians from the French myocarditis study group^23^; (ii) they were positive for EV RNA genome detection; (iii) they were negative for the molecular detection of HHV1-HHV6, PVB19 and adenovirus genomes and for the detection of a bacterial sepsis.

The institutional review committee (Hôpital Européen Georges Pompidou, Paris, France) approved the study, and informed consent was obtained from the patients or subjects’ families at the time of hospitalization or necropsy. Our investigations conformed to the principles outlined in the Declaration of Helsinki for use of human tissue or subjects.

### Cultured cells

HeLa229 cells were grown in minimum essential media (MEM) supplemented by 1% Penicillin–streptomycin (PS) (Gibco, France), 1% L-Glutamine and 10% Fetal Bovin Serum (FBS) (ThermoFisher, France). Human primary CardioMyocytes (HCM, ScienCell Research Laboratories) were cultured in cardiac myocyte medium (CMM) supplemented by 1% Penicillin–streptomycin, 1% Cardiac Myocyte Growth Supplement (CMGS) and 5% FBS (ScienCell Research Laboratories) at 37 °C with 5% CO2. Cardiac myocyte cell cultures were passaged twice a week and the cells were cultured between passages 3 to 9.

### Detection and quantification of viral RNA

Reverse transcription was carried out using Superscript II reverse transcriptase (Invitrogen, Life Technologies). Detection of full-length RNA copy number in heart tissues and peripheral blood samples was quantitated by RT-qPCR using a StepOne plus real time PCR system (ThermoFisher Scientific). Detection of viral RNA was performed using iQ Supermix (BioRad) and 200 nM NC1M primer (nt 456–474) (Fwd: 5′-CCCTGAATGCGGCTAATCC), 200 nM E2 primer (nt 582–601) (Rev: 5′- ATTGTCACCATAAGCAGCCA) and 100 nM S-Ent probe (nt 539–566) (Fwd: 5′- FAM-AACCGACTACTTTGGGTGTCCGTGTTTC-BHQ1) (Supplementary Table S1). RT products were unhybrized at 94 °C for 5 min, and then cDNAs were amplified during 45 cycles as follows: 94 °C for 15 sec, 63 °C for 1 min, and 68 °C for 30 sec. To validate detection and quantitation of EV sequences in clinical samples, serial dilutions of transcripts of wild-type CVB3 (CVB3/28 strain) clones were used as standard for qPCR.

### Phylogenetic analysis

Molecular phylogeny of Enterovirus strains detected in clinical samples was based on the VP1 region nucleotide sequence. Sequences were aligned using Clustal W version 1.81 (www.clustal.org/). Evolutionary distances were calculated using the Kimura 2-parameter method. Trees were constructed using the mixed-linear method (MLM) as implemented in MEGA 6 software^24^. Bootstrap values from 1,000 replicates are shown at the nodes. Scale bar indicates number of nucleotide substitutions per site.

### RT-semi nested PCR

To perform genotyping, viral RNA was extracted from clinical samples (blood samples and heart tissues) using QIAamp Viral RNA mini kit (Qiagen). cDNAs were generated using Superscript II kit (Invitrogen, ThermoFisher) with random hexamers primers (Roche, Life science) in a 20μL reaction mix according the manufacturer’s protocol. cDNAs were then amplified by a two-step semi-nested PCR. Briefly, reverse transcribed products were amplified by KAPA Taq polymerase kit consisting of 1X KAPA TaqA buffer, 200 μM dNTPs, 400 nM of forward primer 222 (Fwd: 5′-CICCIGCIGGIAYRWACAT), 400 nM of reverse primer 224 (Rev: 5′-GCIATGYTIGGIACICAYRT) for the first PCR and 400 nM AN88 (Fwd: 5′-TACTGGACCACCTGGNGGNAYRWACAT), 400 nM AN89 for the second PCR (Rev: 5′- CCAGCACTGACAGCAGYNGARAYNGG), 1U KAPA Taq polymerase in a mix of 25μL with a cycling process composed of one cycle for 1 min at 95 °C, 40 cycles of 30 s at 95 °C, 30 s at 55 °C, 2 min at 72 °C, finishing in a cycle of 2 min at 72 °C. Amplicons were analyzed on Ethidium-bromide stained 1.5% agarose gels in TBE Buffer (Tris–borate-EDTA 10X, Thermo Scientific, ThermoFisher) and DNA bands were gel-purified using QIAquick Gel Extraction Kit (Qiagen) according the manufacturer’s instructions. Purified PCR products were sequenced by Genewiz (Takeley Sanger Sequencing Laboratory, United Kingdom) to detect VP1 gene using AN232 forward primer (5′-CCAGCACTGACAGCA) at 2,602 to 2,616 nucleotide position and AN233 reverse primer (5′- TACTGGACCACCTGG) at 2,977 to 2,963 nucleotide position^25^.

### Rapid amplification of cDNA ends-PCR (RACE PCR)

Total RNA was extracted from heart tissues and peripheral blood samples taken from patients with acute myocarditis using QIAamp Viral RNA mini kit (Qiagen). Viral RNA (200 ng) were reverse transcribed using Superscript II kit (Invitrogen, ThermoFisher) with 400 nM AvCRev (Rev. :5′-AACAGGCGCACAAAGCTACCG)^7^ and was incubated 5 min at 65 °C and then 5 min on ice. The 5′extremity of cDNA was then ligated with 1X T4 DNA ligase buffer (Ambion, ThermoFisher), 50 nM Trp1 DNA adaptor for 5′NCR terminal deletion identification (Fwd: 5′- H-CCTCTCTATGGGCAGTCGGTGAT), 5 units of Ambion T4 DNA Ligase (Ambion, ThermoFisher) was added to the cDNA-mix and was incubated overnight at 16 °C. Positive cDNA were amplified by a classical PCR reaction using KAPA Taq polymerase kit consisting of 1X KAPA TaqA buffer, 200 μM dNTPs, 400 nM AvCRev, 400 nM Trp1 (Fwd: 5′-CCTCTCTATGGGCAGTCGGTGAT), 1U KAPA Taq polymerase in a mix of 25μL with a cycling process composed of one cycle for 3 min at 95 °C, 40 cycles of 30 s at 95 °C, 30 s at 55 °C, 30 s at 72 °C, finishing in a cycle of 30 s at 72 °C. A 2% agarose gel electrophoresis was performed in order to control the length of the PCR products. Amplicons were then sized and quantified by Bioanalyzer High Sensitivity DNA Analyses using Agilent High Sensitivity DNA Kit (Agilent) according to the manufacturer’s instructions. The area of each relevant electrophoresis peak were integrated using software integrated analytic approach (Supplementary Figure S2)^26–28^. The quantification ranges of the RACE-PCR assay followed by a micro-electrophoresis device were assessed using serial tenfold dilutions of synthetic full-length or 5′terminally deleted CVB3 forms in human cardiac or plasmatic total RNA extracts of EV-negative human samples.

### Transfection

2.0 × 10^5^ HCM or HeLa229 cells per well were seeded in 24-well plates (Nunclon delta surface, Thermo Scientific). The plates were incubated overnight at 37 °C. Cells were washed with DPBS (Thermo Scientific) was added. The transfection mixtures (0.1 mL) consisted of 1 µg of synthetic RNA and 1 μl of Lipofectamine 2000 (Thermo Scientific) in Opti-MEM Glutamax. The mixtures were incubated for 20 min at room temperature. Then 100 μl of the mixture was added to the cells, and plates were transferred to a cell incubator at 37 °C for 8 h. The supernatants were collected, and the cells lysed by 500µL of TRI Reagent (Sigma–Aldrich Corporation, Lyon, France).

### Reverse transcription-quantitative PCR using SybrGreen (RT-qPCR)

RNA was extracted from cultured cells with TRI Reagent (Sigma–Aldrich Corporation, Lyon, France) and reverse transcribed using SuperScript II Reverse Transcriptase (RT) (Invitrogen Life Technologies) following to the manufacturer’s instructions. cDNA was subjected to PCR using PowerUp SYBR Green Master Mix (2X) (ThermoFisher Scientific) to detect IFN-β expression. The primers used for PCR detection as follows: for human IFN-β, forward primer AGCTGAAGCAGTTCCAGAAG and reverse primer AGTCTCATTCCAGCCAGTGC. Primers specific to glyceraldehyde-3-phosphate dehydrogenase (GAPDH) (forward primer AGGGCTGCTTTTAACTCTGGT and reverse primer CCCCACTTGATTTTGGAGGGA) was served as the internal control. PCR was carried out in StepOnePlus Real-Time PCR Systems (ThermoFisher Scientific) programmed as follows: 94 °C for 15 s, 63 °C for 10 s, and 72 °C for 15 s for a total of 40 cycles, before melting curve. Results were analyzed by the ΔΔCT method, where CT is threshold cycle, and normalized to GAPDH mRNA. Data are represented as levels of mRNA relative to the mock transfected control samples and are displayed as the means ± SD of results from at least three independent experiments.

### Quantitative ELISA assays for IFN-β

For the quantitative determination of human IFN-β concentrations in supernatants of cultured cells, ELISA assays using commercially available Human IFN-beta DuoSet ELISA kits (R&D Systems, France) were performed according to the manufacturer’s instructions.

### In vitro dephosphorylation assays

The dephosphorylation of synthetics RNAs was carried out using Antarctic phosphatase (10 U), 1X Antarctic Phosphatase buffer (New England BioLabsGmbH, Frankfurt, Germany) and 2 µg of synthetics RNAs in 20 µL final volume for 1 h at 37 °C. Enzymes were deactivated at 80 °C for 2 min. RNAs quality was controlled by loading 8 µL of each controls and dephosphorylation products by electrophoresis on 1% agarose gel.

### Statistical analysis

All statistical analyses were performed using GraphPad Prism 7 (GraphPad) and SAS version 9.4 (SAS Institute Inc.). Quantitative variables were compared using the Mann Whitney U test and a P-value < 0.05 was considered as statistically significant. Linear regression was performed, and slopes were compared using Spearman correlation test. Because selected human biological samples (peripheral blood or heart tissues samples taken from acute myocarditis patients) were of very limited availability and because number was not extendable; we did not have prior data to calculate it and we did not a priori calculate the number of subjects to include in the present study.

### Data availability

We declare that all the data supporting the findings of this study are available within the paper and the Supplementary Information files.

The study materials will not be made available to other researchers for purposes of reproducing the results or replicating the procedure, because the human biological samples (blood and cardiac tissue samples) as well as RNA extracted from HeLa229 and human primary cardiomyocytes used in our experiments remain limited biological sources.

## Supplementary information

Supplementary file1
