# Supplementary material for: Major 5′terminally deleted enterovirus populations modulate type I IFN response in acute myocarditis patients and in human cultured cardiomyocytes
Source: Sci Rep. 2020 Jul 20;10:11947. doi: 10.1038/s41598-020-67648-5 (PMC7371739; doi:10.1038/s41598-020-67648-5)
Supplement: Supplementary file 1 — Supplementary file1 [file 41598_2020_67648_MOESM1_ESM.pdf]

Scientific Reports

Full title:

Major 5'Terminally Deleted Enterovirus Populations Modulate Type I IFN Response in Acute Myocarditis Patients and in Human Cultured Cardiomyocytes

Running title: Interferon Modulation by 5'Terminally Deleted EVB-RNA forms

M. Glenet[1], Y. N'Guyen[1,2], A. Mirand[3,4,5], C. Henquell[3,4,5], A-L. Lebreil[1], F. Berri[1], F. Bani-Sadr[2], B. Lina[6,7,8], I. Schuffenecker[6,7,8], L. Andreoletti*[1,2] and the French Enterovirus Myocarditis Study Group (FEMSG).

[1]University of Reims Champagne-Ardenne and EA4684 Cardiovir research laboratory, Reims, France

[2]Centre Hospitalier Universitaire de Reims, France

[3]National Reference Center of Enterovirus and Parechovirus, Clermont-Ferrand, France

[4]University of Clermont Auvergne, Clermont-Ferrand, France

[5]Centre Hospitalier Universitaire Clermont-Ferrand, France

[6]National Reference Center of Enterovirus and Parechovirus, Lyon, France

[7]Hospices Civils de Lyon, Lyon, France

[8]University of Lyon, Lyon, France

Supplementary data

Tableau S1: Primers used for EV detection, PCR amplification, and sequencing

Primers	Sense	Sequences (5'-3')	Gene	Location (nt)
NC1M	F	CCCTGAATGCGGCTAATCC	5'UTR - Domain V	456-474
S-Ent	F	FAM-AACCGACTACTTTGGGTGTCCGTGTTTC-TAMRA	5'UTR - Domain V	539-566
E2	R	ATTGTCACCATAAGCAGCCA	5'UTR - Domaine VI & VII	582-601
AN232	F	CCAGCACTGACAGCA	VP1	2602-2616
AN233	R	TACTGGACCACCTGG	VP1	2977-2963
222	F	CICCIGCIGGIAYRWACAT	VP1	2969-2951
224	R	GCIATGYTIGGIACICAYRT	VP3	1977-1996
AN88	F	TACTGGACCACCTGGNGGNAYRWACAT	VP1	2977-2951
AN89	R	CCAGCACTGACAGCAGYNGARAYNGG	VP1	2602-2627

Footnotes : F correspond to Forward or sense primers and R correspond to Reverse or antisense primers

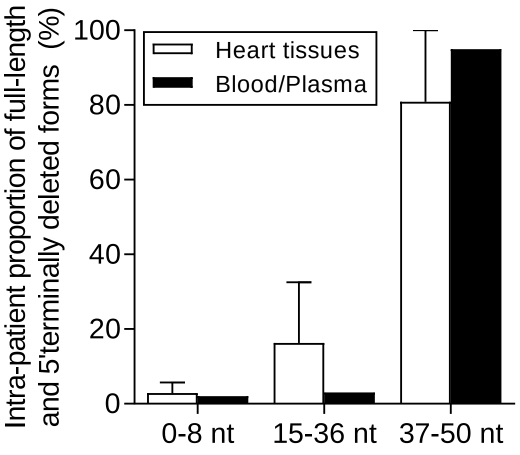

Figure S1: Detection of full-length and 5'terminally deleted EV-B population levels in heart and peripheral blood samples in intra-patient. Comparative analysis of proportion of viral 5'TD (15-36 nt and 37-50 nt forms) and FL form in heart tissues and peripheral blood samples in intra-patient (1 *vs*. 2 samples). Data represent the mean ± SD using Mann-Whitney U test.

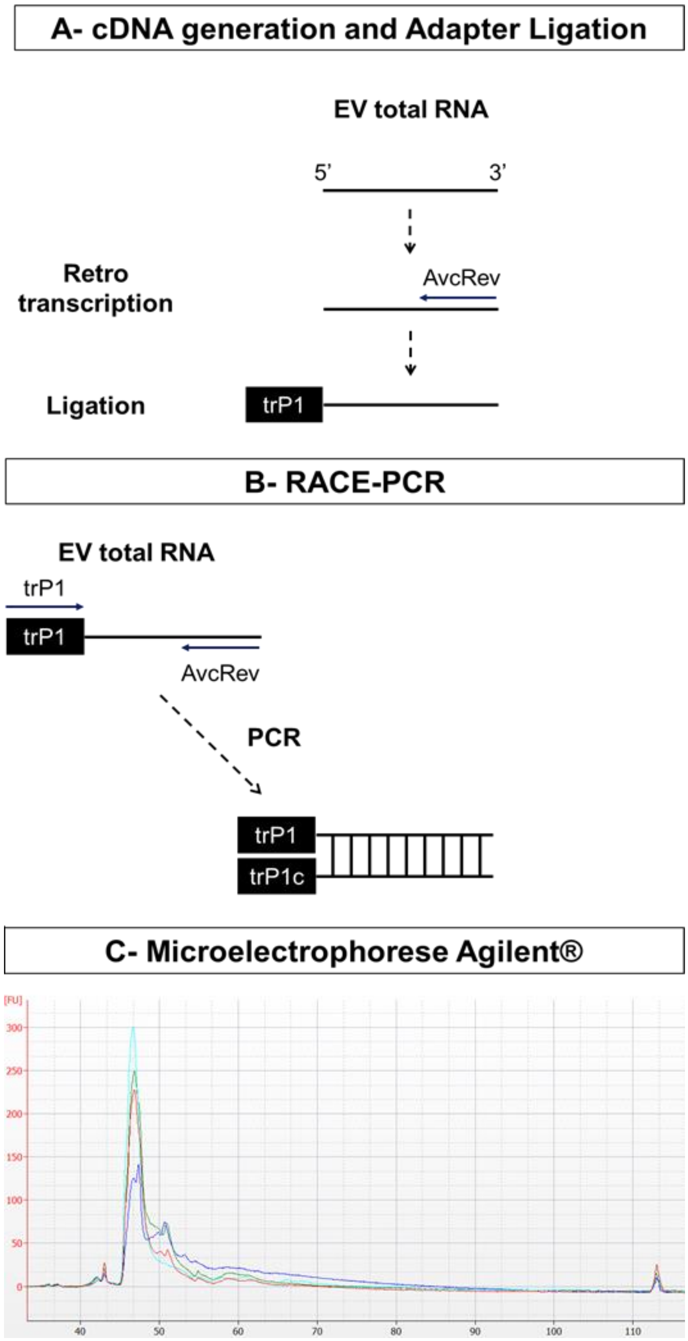

Figure S2: Schematic representation of RACE-PCR following by micro-electrophoresis performed on total nucleic acid extracted from heart tissues and peripheral blood samples of acute myocarditis patients. **A.** cDNA generation and adapter ligation: a retro transcription step was performed by using AvcRev primer and allowed the generation of a cDNA of the 5′ non-coding region until the terminal. TrP1 adapter was then ligated to the 5′ extremity of cDNA generated. **B.** Rapid amplification of cDNA ends PCR (RACE): a classical PCR using primers trP1 and AvcRev was performed on cDNA generated from cDNA ligation and enabled the amplification of the 5′ terminal genomic region. The length of the amplicons was assessed by 2% agarose gel electrophoresis. **C.** Micro-electrophorese Agilent: DNA chip containing micro-channels allowing nucleic acid fragments bases separation after electrophoresis. Application of Agilent High Sensitivity DNA kit for sizing and quantitation of DNA samples. EV, enterovirus

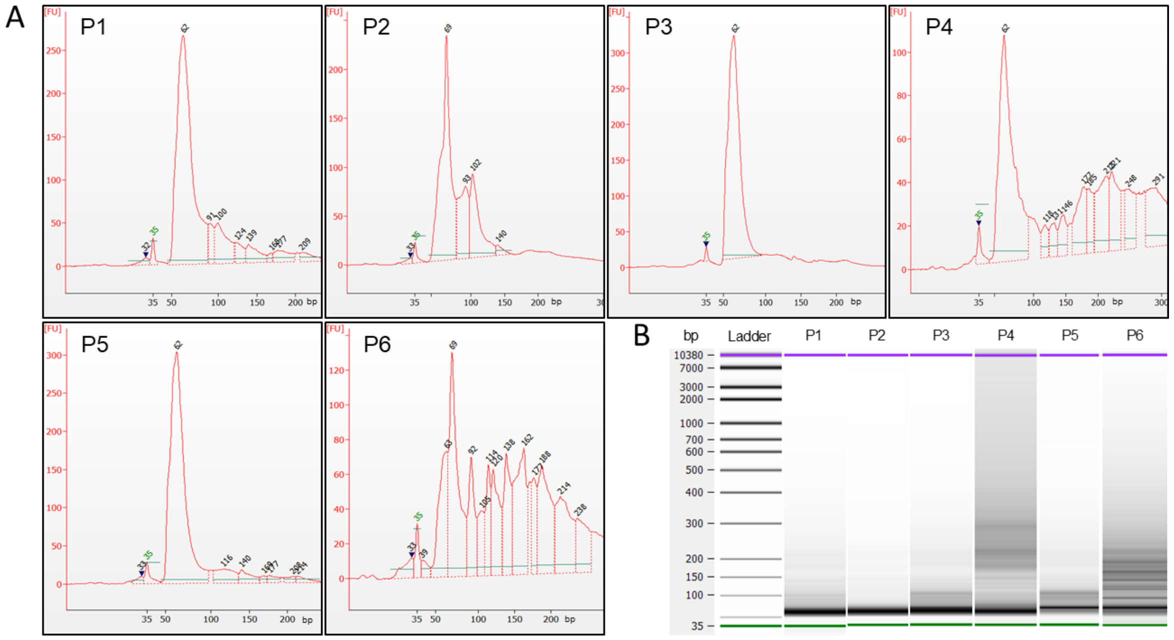

Figure S3: Identification of full-length and EV-B TD forms in peripheral blood samples of acute myocarditis patients. **A.** Electrophoregrams of isolated 5’end viral cDNA detected from patient 1 to 6 corresponding to the acute myocarditis study cases. **B.** The image swhows a digital eletrophoregram obtained from Agilent bioanalyzer. The lane 1 shows DNA ladder. Lanes 2 to 6 correspond to patients 1 to 6 suffering from acute myocarditis.

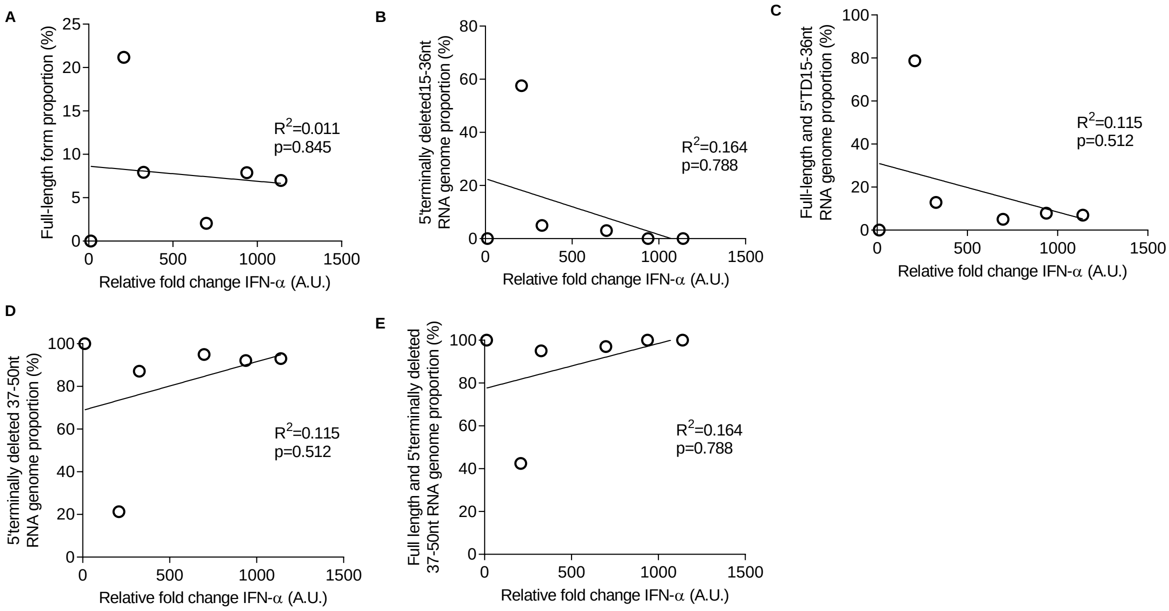

Figure S4: Relationships between EV-B full-length or identified EV-B 5'terminally deleted population proportions and IFN-α level. **A.** FL form proportion (%) correlation between relative fold change of IFN-α (A.U.) (n=6). **B.** 5'terminally deleted 15-36nt RNA genome proportion (%) correlation between relative fold change of IFN-α (A.U.) (n=6). **C.** Full-length and 5'terminally deleted 15-36nt RNA genome proportion (%) correlation between relative fold change of IFN-α (A.U.) (n=6). **D.** 5'terminally deleted 37-50nt RNA genome proportion (%) correlation between relative fold change of IFN-α (A.U.) (n=6). **E.** Full-length and 5'terminally deleted 37-50nt RNA genome proportion (%) correlation between relative fold change of IFN-α (A.U.) (n=6). Linear regression were performed, and slopes were compared using Spearman test. FL, full-length; TD, terminally deleted.
